# Magnesium Enhances Exercise Performance via Increasing Glucose Availability in the Blood, Muscle, and Brain during Exercise

**DOI:** 10.1371/journal.pone.0085486

**Published:** 2014-01-20

**Authors:** Hsuan-Ying Chen, Fu-Chou Cheng, Huan-Chuan Pan, Jaw-Cheng Hsu, Ming-Fu Wang

**Affiliations:** 1 Department of Food and Nutrition, Providence University, Shalu District, Taichung, Taiwan; 2 Stem Cell Center, Department of Medical Research, Taichung Veterans General Hospital, Taichung, Taiwan; 3 Department of Applied Chemistry, Providence University, Shalu District, Taichung, Taiwan; 4 Department of Neurosurgery, Taichung Veterans General Hospital, Taichung, Taiwan; 5 Department of Applied Cosmetology & Graduate Institute of Cosmetic Science, HungKuang University, Shalu District, Taichung, Taiwan; Universidad Pablo de Olavide, Centro Andaluz de Biología del Desarrollo-CSIC, Spain

## Abstract

Glucose mobilization and utilization in the periphery and central nervous system are important during exercise and are responsible for exercise efficacy. Magnesium (Mg) is involved in energy production and plays a role in exercise performance. This study aimed to explore the effects of Mg on the dynamic changes in glucose and lactate levels in the muscle, blood and brain of exercising rats using a combination of auto-blood sampling and microdialysis. Sprague-Dawley rats were pretreated with saline or magnesium sulfate (MgSO_4_, 90 mg/kg, i.p.) 30 min before treadmill exercise (20 m/min for 60 min). Our results indicated that the muscle, blood, and brain glucose levels immediately increased during exercise, and then gradually decreased to near basal levels in the recovery periods of both groups. These glucose levels were significantly enhanced to approximately two-fold (*P*<0.05) in the Mg group. Lactate levels in the muscle, blood, and brain rapidly and significantly increased in both groups during exercise, and brain lactate levels in the Mg group further elevated (*P*<0.05) than those in the control group during exercise. Lactate levels significantly decreased after exercise in both groups. In conclusion, Mg enhanced glucose availability in the peripheral and central systems, and increased lactate clearance in the muscle during exercise.

## Introduction

Glucose is the major energy source in cells, and glucose mobilization in the circulatory system and local body systemsduring exercise is postulated to involve a complex mechanism [1]. Magnesium (Mg) plays a central role in glucose utilization and metabolism, but exercise may result in Mg deficiency because of increased Mg excretion in sweat and urine [2]. Accumulating evidence indicates that Mg improves exercise performance, but the effects of Mg intervention during exercise on whole-body glucose mobilization remain unclear [3], [4].

Previous studies have revealed that hyperglycemia may occur during short-term, high intensity-exercise, but prolonged or exhausting exercise may induce hypoglycemia [5]–[7]. In general, muscle glycogen is the major fuel that rapidly depletes during the acute phase of exercise [8], [9]. When muscle glycogen is depleted, blood vessels carry nutrients, including glucose, to working muscles to support exercise continuity. Furthermore, the brain is a heavy energy consumer, and plays a decisive role in the regulation of whole body energy metabolism [10]. Brain glucose utilization increases in response to motor tasks and stimulation of visual, auditory, olfactory and somatosensory cells [11]–[14]. However, glucose availability and utilization in the brain during exercise remain controversial. In a study by Bequet et al. and in our previous studies, brain glucose concentrations increased during exercise [15], [16]. In contrast, brain glucose concentrations remained unchanged during bicycle exercise [17], and decreased with increasing exercise intensity [18].

Magnesium (Mg) is the second most abundant intracellular cation and serves as a co-factor in more than 300 enzymatic reactions, including energy production [19]. Mg is involved in glucose metabolism and enhances exercise performance. In general, long-term exercise increases Mg excretion through sweat and urine and may result in Mg deficiency [20]. Therefore, exercise performance is highly dependent on the regulation and maintenance of Mg homeostasis. Moreover, exercise performance appears to be impaired under Mg deficiency conditions [21]–[23]. Mg supplementation improves exercise performance in forced swimming and treadmill exercises [24], [25]. Therefore, the enhancement of exercise performance by Mg could be related to glucose availability and regulation. In general, exercise needs the integration of several systems in the whole body. For example, the muscle-skeletal system responds to the action and the circulatory system needs to increase the cardiac output for supporting more oxygen and other related compounds. Brain and spinal cord control, plan, and regulate the motor commands. Several studies have investigated glucose changes only in the blood, muscle, or brain to study systemic effects of exercise [15], [26]–[29]. Therefore, exploring the glucose changes in the blood, muscle, and brain simultaneously is important to understand the systemic changes. In addition, effects of Mg on the dynamic glucose changes in the blood, muscle, and brain remain unclear and at times controversial. Furthermore, obtaining samples from multiple locations in an exercising animal is always an analytical challenging.

Microdialysis and repeated blood sampling techniques have been previous developed by us [24], [25] and others [26]–[29]. In the present study, we combined an auto-blood sampling system with two microdialysis systems to determine the effects of Mg on the dynamic changes in glucose and lactate concentrations in the blood, muscle, and brain simultaneously in exercising rats.

## Materials and Methods

### Ethics Statement

Animal care and experimental procedures were approved (Permit Number: La-98657) by the Institutional Animal Care and Use Committee (IACUC) of Taichung Veterans General Hospital. All data required according to the ARRIVE guidelines were included [30]. All surgeries were performed under isoflurane anaesthesia, and all efforts were made to minimize suffering.

### Animal

**Figure 1:**
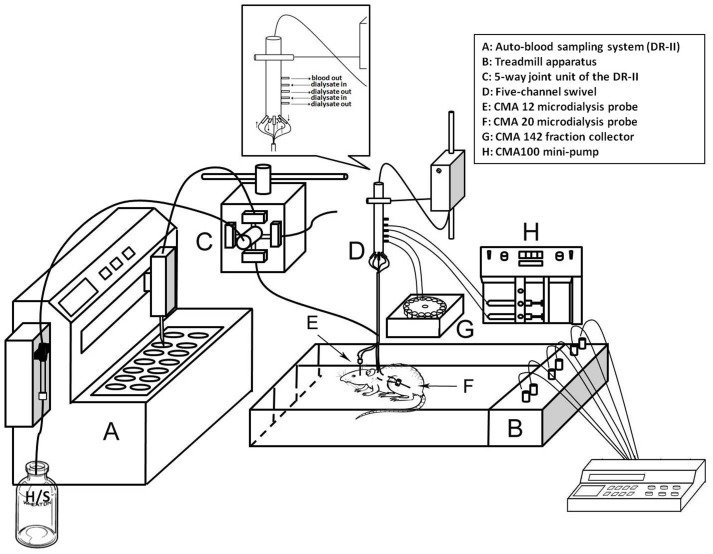
Schematic of a rat on the exercise treadmill with a combined auto-blood sampling and microdialysis systems.

Adult male 8-weeks-old Sprague-Dawley (SD) rats weighing between 300–350 g were purchased from BioLASCO Taiwan Co., Ltd. (Taipei, Taiwan). All rats were housed in a temperature (25°C) and light-controlled room (12∶12 h light-dark cycle), standard rat chow, and water ad libitum. The rats were randomly assigned to the control and Mg groups. Rats were pretreated with saline or MgSO_4_ (90 mg/kg, i.p.) 30 min before treadmill exercise (20 m/min for 60 min). The illustration of the treadmill device and apparatus for blood and microdialysis sampling during exercise is described in Figure 1.

### Method Validation

Sampling calibrations, microdialysis probes in vitro recovery test, and microdialysis analyzer calibrations were performed according to the manufacturers’ instructions prior to all experiments. In vitro recovery of each probe was determined and approved prior to experiments. The recovery of a microdialysis probe less than 15% was excluded. Coefficient of variation (CV) represents the ratio of the standard deviation (SD) to the mean in regard to the precision of sampling volumes and data analysis. The CV values of blood and microdialysis sampling volumes were less than 5%. The intra-assay precision and stability of all standard mixture concentrations were also measured, and their CV values were less than 3%.

### Treadmill Exercise Protocol

Each rat was habituated to the treadmill apparatus (Model T306, Diagnostic & Research Instruments Co., Taoyuan, Taiwan) in the experiment (Figure 1). According to the experimental procedure, after a 60-min rest period, each rat was forced to exercise for 60 min at a speed of 20 m/min, and then allowed to recover for an additional 180 min.

### Auto-Blood Sampling System (DR-II)

Prior to all experiments, a catheter (TPC/40, EICOM, Kyoto, Japan) was cannulated into the jugular vein. To prevent the TPC/40 catheter from being damaged by the animal, the catheter was tunneled under the skin to the nape of the neck, as previously described [25].

Blood samples (50 µl) were automatically collected via an implanted catheter in the jugular vein every 15 min during the rest period, the treadmill exercise, and the additional 180 min recovery periods using a computer-aided auto-blood sampling system (DR-II, EICOM, Kyoto, Japan), as previously described [25]. Blood samples were loaded in a tube containing heparin solution to prevent coagulation, and subsequently centrifuged at 800 × g for 10 min at 4°C. Afterward, glucose and lactate concentrations were determined using a CMA/600 microdialysis analyzer (CMA, Stockholm, Sweden).

### Brain and Muscle Microdialysis Samplings

A CMA/12 guide cannula (CMA, Stockholm, Sweden) was inserted in the right striatum (AP = 0.2 mm, ML = 3.0 mm, DV = 3.2 mm from the bregma) using a stereotaxic instrument. The cannula was fixed with two screws and dental cement. After surgery, animals were allowed 1–2 days to recover. A CMA/12 probe (CMA/12 Elite 14/04 PAES, Stockholm, Sweden) was implanted through the CMA/12 guide cannula into the striatum of a rat on the experimental day.

On the experimental day, a small incision in the skin over the right biceps femoris was made, and a microdialysis probe introducer was inserted into the muscle. subsequently, a CMA/20 microdialysis probe (CMA/20 Elite 20/04 PAES, Stockholm, Sweden) was inserted through the introducer, and secured by suturing both sides of the wings on the muscle.

The muscle and brain microdialysis probes were perfused with Ringer’s solution at a rate of 2 µl/min allowing collection of 30 µl samples every 15 min. The total collection time (5.5 h) of the study included a 90-min rest period (including an injection of magnesium sulfate or saline), 60-min exercise, and 180-min recovery periods. The CMA600 microdialysis analyzer (CMA, Stockholm, Sweden) was used for determination of glucose and lactate concentrations of dialysates [24].

### Statistical Analysis

All data are expressed as mean ± SEM. Mann-Whitney test and Wilcoxon Signed-Ranks test were performed for comparision between and within groups. All analyses were performed using the Statistical Package for the Social Sciences software (SPSS 14.0). Differences were considered statistically significant at *P*<0.05.

## Results

### Glucose Changes in the Blood, Muscle, and Brain

**Figure 2:**
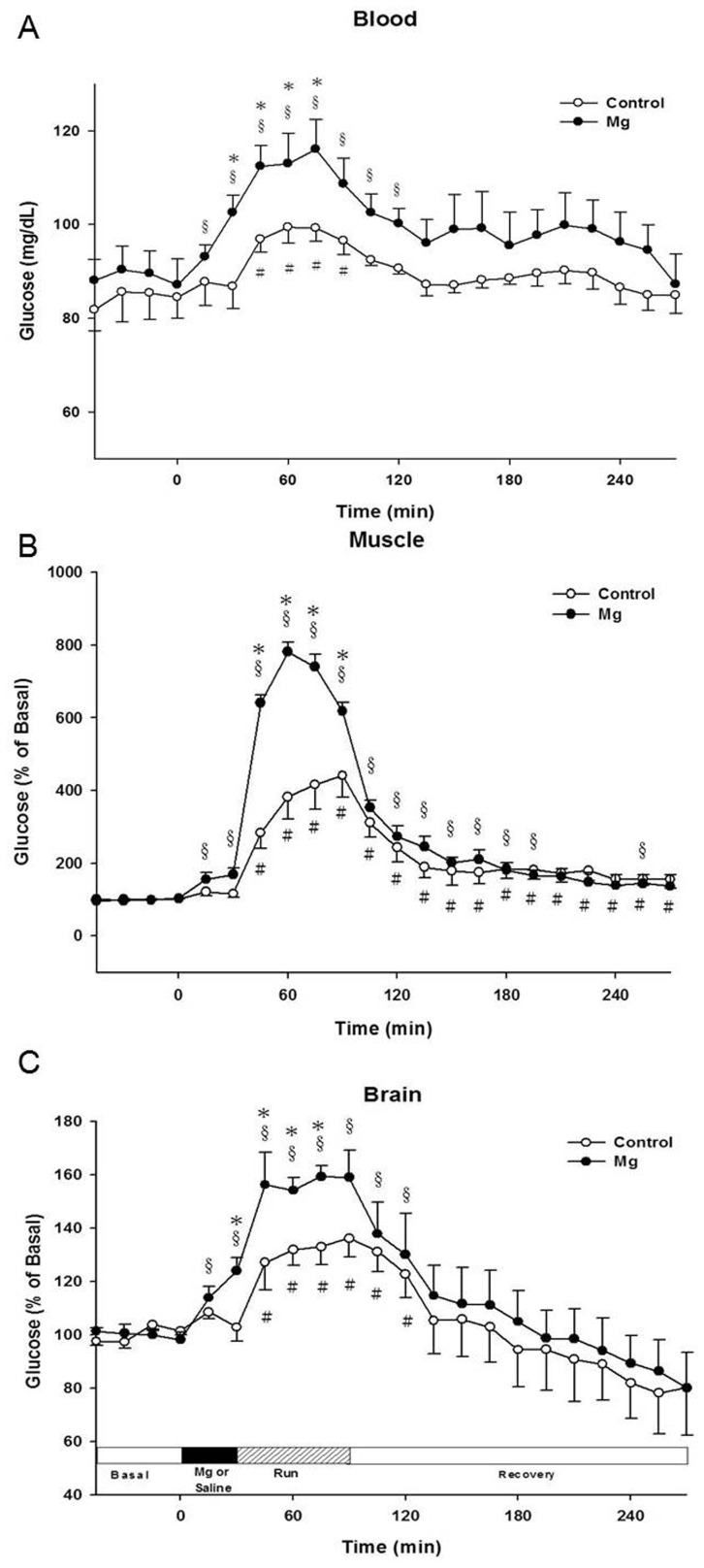
Time profiles of the effect of Mg on the changes in glucose concentrations in the (A): blood, (B): muscle, and (C): brain. * *P*<0.05 compared with the control group, (Mann-Whitney test); # *P*<0.05 compared with the basal levels in the control group, (Wilcoxon Signed Ranks Test); § *P*<0.05 compared with the basal levels in the Mg group, (Wilcoxon Signed Ranks Test).

The basal concentrations of muscle, blood and brain glucose were shown in Table 1. Blood glucose concentrations in the control group slightly rose to approximately 90 mg/dL after saline administration, and then increased to approximately 100 mg/dL during exercise (Figure 2-A). After exercise, glucose slowly decreased to the basal level in the recovery periods. In the Mg group, glucose increased to approximately 95 mg/dL (*P*<0.05) after Mg administration, and then rapidly increased to approximately 110 mg/dL after for 15 min of exercise (*P*<0.05). Glucose then gradually returned to approximately 95 mg/dL the same as the basal level, and remained at this level throughout the recovery period.

**Table 1:** Mean ± SEM basal levels of glucose and lactate concentrations in the blood, muscle, and brain in control and magnesium (Mg) groups.

	Blood		Muscle		Brain	
	Control (mg/dL)	Mg (mg/dL)	Control (µM)	Mg (µM)	Control (µM)	Mg (µM)
Glucose	84.5±4.5	87.1±5.6	613±40	618±20	225±13	208±15
Lactate	9.7±1.6	10.9±1.2	157±20	155±12	127±9	123±13

Muscle glucose concentrations in the control group, slightly increased immediately after saline was given, then increased to approximately 300%–440% of the basal levels during exercise. In the Mg groups, muscle glucose immediately increased (approximately 160%–170%) within 15 min following Mg administration. In the exercise period, glucose increased markedly to 650%–780% of the basal level (*P*<0.05). After exercise, glucose concentrations in both groups were diminished to approximately 200% of the basal level within 15 to 30 min during the recovery period (Figure 2-B).

Brain glucose concentrations in the control groups increased to 130%–140% of the basal level (*P*<0.05) during the exercise period, and then diminished to 120% of the basal level after exercise. Glucose slowly returned to approximately the basal level 45 min after exercise (Figure 2-C) in the control group. In the Mg group, brain glucose increased to 110%–125% immediately after Mg administration for 15 min (*P*<0.05). Brain glucose was increased to approximately160%, and then plateaued during exercise. During recovery periods, glucose gradually returned to the basal level in the Mg group.

### Lactate Changes in the Blood, Muscle, and Brain

**Figure 3:**
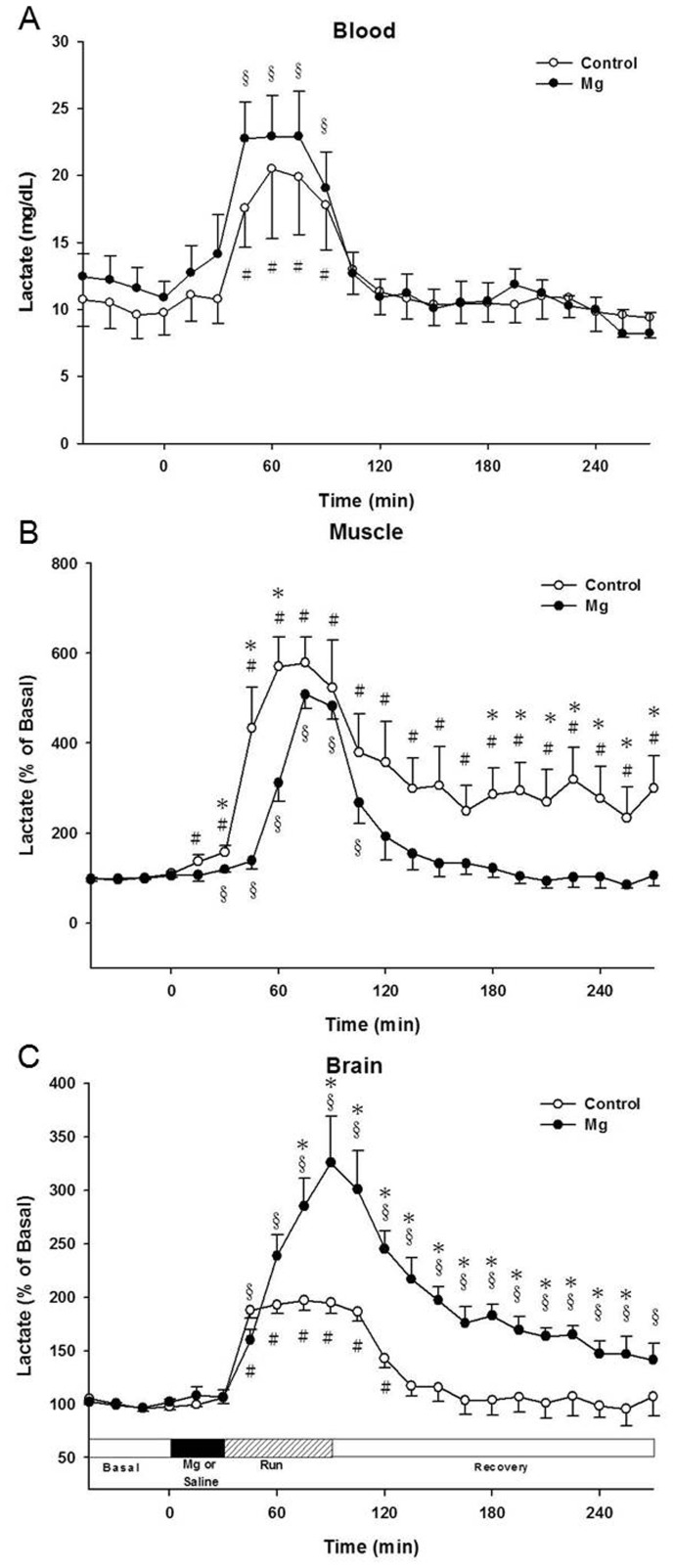
Time profiles of the effect of Mg on the changes in lactate concentrations in the (A): blood, (B): muscle, and (C): brain. * *P*<0.05 compared with the control group, (Mann-Whitney test); # *P*<0.05 compared with the basal levels in the control group, (Wilcoxon Signed Ranks Test); § *P*<0.05 compared with the basal levels in the Mg group, (Wilcoxon Signed Ranks Test).

The basal concentrations of muscle, blood and brain lactate were shown in Table 1. Blood lactate concentrations in the control group immediately increased to approximately 15–20 mg/dL compared with the basal level during exercise (*P*<0.05), then gradually decreased to approximately the basal levels, approximately 60 min after exercise, and remained the same until the end of the recovery period (Figure 3-A). The blood lactate profile of the Mg group was similar to that of the control group during the whole experimental period.

Muscle lactate concentrations in the control group sharply rose to approximately 430% of the basal level and reached approximately 600% of the basal level at the beginning of exercise, whereas lactate returned to 360% of the basal level within 30 min after exercise. Lactate then remained at 300% of the basal level and was significantly higher even at the end of the experiment (*P*<0.05) (Figure 3-B). In the Mg group, the increase in lactate was delayed by 45 min of compared with the control group after the onset exercise, before reaching the peak (approximately 500% of the basal level). Lactate rapidly diminished to approximately 260% of the basal level at the beginning of the recovery period, and then gradually declined to basal levels.

During exercise, brain lactate concentrations in the control group were significantly increased to approximately 200% of the basal level, and slowly diminished to the basal levels during the recovery periods (Figure 3-C). In the Mg group, brain lactate concentrations rose to approximately 190% of the basal levels and reached a peak of approximately 320% of the basal levels during exercise. In the recovery period, lactate gradually diminished to approximately 200% of the basal levels approximately 60 min after exercise. Brain lactate concentrations of the Mg group were approximately 200% higher than those of the control group (*P*<0.05).

## Discussion

Mg administration significantly enhanced the availability of glucose in the blood, muscle, and brain, but diminished the accumulation of lactate concentrations in blood and muscle in exercise. The measurements were obtained simultaneously using an auto-blood sampling system (DR-II) combined with two microdialysis systems to determine the dynamic glucose changes simultaneously in the blood, muscle, and brain (Figure 1). Moreover, all in vitro and in vivo assays in the present study were validated and shown to be reliable within 5%.

### Glucose in Blood, Muscle, and Brain

Exercise elevates whole body glucose production and utilization to meet fuel demands. In general, muscle glycogen is the major fuel and rapidly depletes during the acute phase of exercise [8], [9]. At that time, liver glycogen may break down to glucose as an alternative energy source, and then blood flow carries nutrients, including glucose, to working muscles supporting exercise. The rates of glycogen deplete with increasing exercise intensity [31]. Thus, blood glucose concentrations increased 15% in our study during exercise and it doubled (30% increase) in the Mg group (Figure 2-A). Muscle glucose concentrations rapidly increased to approximately 400% of the basal level, and further rose to approximately 800% of the basal level at the beginning of exercise in the Mg group (Figure 2-B). During exercise, two-fold higher glucose concentrations were detected in the blood, muscle, and brain, in the Mg group compared with those of the control group.

Glucose is transported to muscle via peripheral blood vessels for fulfilling the energy requirement during exercise. It has been established that blood glucose is transported to muscle premarily by the glucose transporter-4 (GLUT-4) [32]. GLUT-4 may be hyper-activated after exercise in order to fulfill the need to increase utilization of glucose in the working muscle (and also possible in brain) for further glycolysis [33]. During Mg deficiency, GLUT-4 protein expression is decreased, in addition, Mg modulates insulin activity for further glucose transported by GLUT-4 [34], [35]. Therefore, in the present study, Mg pretreatment could improve GLUT-4 protein expression, allowing more glucose transport to the muscles during exercise. Energy supply in the brain during exercise is critical; brain glucose concentrations increased compared with the basal levels in both groups (Figure 2-C). Neurons in the require energy substrates for the production of secretory factors to coordinate motor movement, heart rates, and blood pressure during exercise [29]. Of course, glucose is one of the obligatory energy substrates in the brain. Increased brain glucose levels enhance the exercise performance after Mg administration in a swimming exercise model [16], [25]. Thus, increasing brain glucose levels are beneficial during exercise. Exercise could be result in a significant increase in blood brain barrier (BBB) permeability, leading to greater uptake of blood glucose from the blood compartment into the brain [36], [37]. In addition, glucose transporter -3 (GLUT-3) is activated increasing the transport of blood glucose following exercise and GLUT-3expression is up-regulated with increased blood glucose concentrations [38]. GLUT-3 mediates the transport of glucose into the neurons and glia for energy utilization, and is highly expressed in brain [39], [40]. Mg has also been documented to enhance GLUT-3 protein expression [38], [41], which is consistent with the notable increases in brain glucose concentrations in the Mg group compared with those in the control group (Figure 2-C) in the present study. In general, the brain is capable of producing substrates to coordinate motor functions during exercise [29], [36]. For example, epinephrine increases glycogenolysis and gluconeogenesis at the liver, reduces insulin secretion while increasing glucagon release from the pancreatic islets, reduces glucose uptake and utilization and increases glycolysis by muscle, and increases lipolysis in adipose tissues and other organs. However, further study is needed to understand the regulation and coordination mechanisms contributing to brain substrates in exercise [29].

The km values (an indicator of the affinity of the transporter protein for glucose molecules) of GLUT-3 and GLUT-4 are 1–1.6 mM (18–28.8 mg/dL) and 4.3–4.6 mM (77.4–82.8 mg/dL), respectively [42]–[45]. These transporters facilitate the transportation of glucose when the glucose concentrations reached the respective km levels. The basal concentrations of glucose in the brain and muscle obtained from the microdialysis technique were approximately 5%–20% of the basal level, depending on the in vivo recovery of probe. The recovery rate is also dependent on the rate of perfusion, dialysis membrane types, and detected conditions. An in vitro study indicated that the recovery rate was higher when the perfusion rate was lower. Thus, microdialysis data have been presented as % of the basal level in conventional manners [46]. On the other hand, to clarify the actual glucose levels in the muscle and brain during exercise requires sacrificing the animals at different time points, and thus limits observing continuous glucose level changes. Microdialysis techniques provide the advantages of continuous sampling, and analysis of dynamic changes in analyte concentrations (e.g., glucose and lactate) in relatively small volumes of dialysates. The interactions of glucose in the muscle and brain with glucose transporters remains unclear. We may speculate that GLUT-3 activity increase after Mg administration from previous studies [38], [41]. Furthermore, long term and intense exercise may result in Mg deficiency which impairs insulin receptor activity [35], [47]. Lower Mg concentrations may affect insulin secretion and insulin receptor activity, and have been shown to down-regulate Mg transporter gene (TRPM6) expression [48]. TRPM6 reabsorbs Mg, mainly in kidney, and plays a pivotal role in Mg homeostasis [49]. Thus, Mg supplementation may provide an additional source of glucose transport into cells via increasing BBB permeability, as well as up-regulating GLUT-3, GLUT-4, and TRPM6 transporters genes expressions. Hence, increasing glucose levels in the blood, muscle, and brain were observed after Mg supplementation. Further investigation is needed to explore these mechanisms.

### Lactate Changes in the Blood, Muscle, and Brain

Our data also revealed that maximal muscle lactate accumulation was significantly delayed during the first episode of exercise in the Mg group (Figure 3-B). This may also postpone peripheral fatigue under certain conditions. Muscle lactate concentrations significantly increase to a peak in response to a short term, intense treadmill exercise [50]. However, Mg delayed the rise of lactate concentrations to the peak for approximately 45 min during the same exercise intensity. Lactate concentrations rapidly diminished after exercise and returned to the basal level during the recovery period in the Mg group (Figure 3-B). Nevertheless, muscle lactate concentrations remained approximately 300% of the basal level during the 3-hour recovery period in the control group.

Fatigue is one of the most important factors in impairment of exercise performance. Fatigue is associated with exercise involving both peripheral and central fatigues. It is commonly accepted that muscle lactate accumulation is one of the causes of peripheral fatigue, reducing the efficiency of muscular contraction and exercise performance. Muscle lactate is transported into blood via monocarboxylate transporters (MCTs) and may be converted to glucose later via the Cori cycle in the liver [51], [52]. The additional glucose is then returned to the blood vessels for continuous use by the muscle as an additional energy source. Furthermore, Mg involves the synthesis of ATP by activating adenosine triphosphate and contributes to the fundamental energy supply. Our data demonstrate that muscle lactate accumulation is delayed (Figure 3-B) and higher blood glucose concentrations (Figure 2-A) are available after Mg administration in exercising rats. This may suggest Mg enhances exercise performance by activating the transportation of glucose or increasing glucose available in the blood, muscle and brain.

Central fatigue is thought to be associated to an insufficient energy supply, involving various substrates secreted within the brain. In our data, brain lactate was significantly increased during and after exercise, especially in the Mg group (Figure 2-C). Mg may enhance exercise performance by means of increasing lactate molecules via one or more as yet unknown mechanisms. Lactate is a metabolite of glucose, and it has been considered a metabolic waste in the past. However, there is clear evidence indicating lactate can be utilized as an adequate energy substrate for brain tissues [53], [54]. Lactate has been postulated to act as a pseudo-hormone (as well as cell signaling substrate) which can influence the delivery of oxidative and gluconeogenic substrates [55]. However, to respond to sudden increases in energy required during exercise, astrocytes break down glycogen to lactate, and export it to neurons as a fuel to support axonal functions when glucose supply is inadequate or unavailable [56], [57]. Mg may enhance the expression of the astrocyte-neuron lactate shuttle by increasing BBB permeability, which may account for the further increase in brain lactate in the Mg group.

### Techniques Overcome Repeated Sampling Limitation

In sport science research, repeated blood samples were obtained from the tail vein or using retro-orbital puncture before and after exercise [58]. However, these techniques cannot be done repeatedly within a short period in the same animal. These conventional techniques are associated with stressful behavioral responses during blood sampling [59], [60]. Moreover, these stressful behavioral responses and changes, measured before or after exercise, may not represent their actual dynamic changes during exercise. Therefore, the use of a silicone catheter implanted into the jugular vein, and sampling by an auto- sampling system (DR-II) may resolve this experimental problem. In addition, microdialysis is a suitable technique allowing continuous sampling from the extracellular space of brain and muscle tissues in free-moving animals [61]. In the present study, the auto-blood sampling system was combined with microdialysis techniques to determine the glucose and lactate concentrations simultaneously in the blood, muscle, and brain during exercise. To the best of our knowledge, this is the first experiment to combine these techniques in an exercising rat model to investigate dynamic changes of glucose and lactate levels multiple locations in sport science.

## Conclusion

Our data demonstrate that Mg possibly enables the provision of an adequate glucose source by increasing glucose availability and facilitating the clearance of lactate. An integral system for the simultaneous determination of dynamic changes in glucose and lactate in the blood, muscle, and brain of exercising rats was established. Further research is needed to elucidate the mechanisms involved by exploring the regulation of Mg and glucose transporters during exercise. In addition, the newly developed technique described herein may allow for a better understanding of continuous changes in any other candidate compounds in the peripheral and central systems using animal models.
